# Supplementary material for: Exogenous Serotonin (5-HT) Promotes Mesocotyl and Coleoptile Elongation in Maize Seedlings under Deep-Seeding Stress through Enhancing Auxin Accumulation and Inhibiting Lignin Formation
Source: Int J Mol Sci. 2023 Dec 2;24(23):17061. doi: 10.3390/ijms242317061 (PMC10707020; doi:10.3390/ijms242317061)
Supplement: Supplementary file 1 [file ijms-24-17061-s001.zip › ijms-2701912-supplementary.pdf]

Table S1. Mitigation effect index (MEI) of all traits in ly119-2 seedlings.

**MEI (Trait)**	**ly119–2**					
	**T1**	**T2**	**T3**	**T4**	**T5**	**CV (%)**
MEI (Mesocotyl length)	0.157	0.137	0.608	1.118	0.039	109.72
EI (Coleoptile length)	0.700	1.200	1.500	2.400	1.300	43.81
MEI (Mesocotyl and coleoptile total length)	0.246	0.311	0.590	1.033	0.246	69.58
MEI (Mesocotyl-to-coleoptile ratio)	–0.117	–0.286	–0.150	–0.133	–0.391	55.21
MEI (Mesocotyl coarse)	1.736	1.483	2.195	1.529	2.046	17.47
MEI (Coleoptile coarse)	2.111	2.500	3.204	1.796	3.667	29.07
MEI (Seedling length)	0.600	0.273	1.164	1.636	1.418	55.84
MEI (Seedling weight)	0.171	–0.326	0.813	1.039	0.908	111.23
MEI (Mesocotyl weight)	0.906	0.503	1.133	1.136	0.181	54.30
MEI (Coleoptile weight)	2.294	5.711	10.965	9.871	15.565	57.26
MEI (Seeding emergence rate)	0.000	0.050	0.600	0.950	0.300	104.69
MEI (IAA content in mesocotyl)	0.608	0.378	1.040	2.540	0.175	99.79
MEI (IAA content in coleoptile)	0.838	2.235	2.426	3.000	2.059	37.65
MEI (H_2O_2 content in mesocotyl)	0.194	0.215	0.500	1.048	0.098	94.01
MEI (H_2O_2 content in coleoptile)	0.271	0.449	1.063	0.821	0.562	49.32
MEI (Lignin content in mesocotyl)	0.592	0.469	1.184	2.041	0.163	83.46
MEI (Lignin content in coleoptile)	0.392	0.536	0.505	0.948	0.412	40.45

CK(+): 3 cm seeding depth + 0 mg/L serotonin (5-HT) application, CK(-): 20 cm seeding depth + 0 mg/L 5-HT application, T1: 20 cm seeding depth + 1.0 mg/L 5-HT application, T2: 20 cm seeding depth + 1.5 mg/L 5-HT application, T3: 20 cm seeding depth + 2.0 mg/L 5-HT application, T4: 20 cm seeding depth + 2.5 mg/L 5-HT application, T5: 20 cm seeding depth + 3.0 mg/L 5-HT application. CV (%): coefficient of variation. H_2O_2: hydrogen peroxide; IAA: indole-3-acetic acid. CV: coefficient of variation.

Table S2. Mitigation effect index (MEI) all traits in ZRX87-1 seedlings.

**MEI (Trait)**	**ZRX87–1**					
	**T1**	**T2**	**T3**	**T4**	**T5**	**CV (%)**
MEI (Mesocotyl length)	0.141	0.109	0.598	0.793	–0.228	144.92
MEI (Coleoptile length)	2.167	4.000	17.000	18.667	2.333	93.60
MEI (Mesocotyl and coleoptile total length)	0.265	0.347	1.602	1.888	–0.071	108.79
MEI (Mesocotyl-to-coleoptile ratio)	–0.096	–0.275	–0.740	–0.699	–0.411	62.00
MEI (Mesocotyl coarse)	0.530	0.365	0.641	0.398	0.044	56.92
MEI (Coleoptile coarse)	0.971	1.049	0.473	0.778	0.481	35.77
MEI (Seedling length)	0.523	0.826	1.965	2.326	0.512	69.44
MEI (Seedling weight)	0.309	0.747	2.941	2.962	–0.205	110.02
MEI (Mesocotyl weight)	1.645	2.909	5.573	7.371	0.692	76.35
MEI (Coleoptile weight)	0.303	1.876	4.469	4.475	–2.035	153.73
MEI (Seeding emergence rate)	0.000	0.000	0.150	0.500	0.000	166.77
MEI (IAA content in mesocotyl)	0.108	–0.026	0.508	1.013	–0.521	266.85
MEI (IAA content in coleoptile)	0.500	0.707	1.879	3.138	0.103	97.85
MEI (H_2O_2 content in mesocotyl)	0.226	0.152	0.154	0.271	–0.702	2014.10
MEI (H_2O_2 content in coleoptile)	0.069	0.333	0.900	1.034	0.060	96.21
MEI (Lignin content in mesocotyl)	0.283	0.087	0.739	1.413	0.413	88.44
MEI (Lignin content in coleoptile)	1.500	3.000	11.375	13.250	1.625	92.60

CK(+): 3 cm seeding depth + 0 mg/L serotonin (5-HT) application, CK(-): 20 cm seeding depth + 0 mg/L 5-HT application, T1: 20 cm seeding depth + 1.0 mg/L 5-HT application, T2: 20 cm seeding depth + 1.5 mg/L 5-HT application, T3: 20 cm seeding depth + 2.0 mg/L 5-HT application, T4: 20 cm seeding depth + 2.5 mg/L 5-HT application, T5: 20 cm seeding depth + 3.0 mg/L 5-HT application. CV (%): coefficient of variation. H_2O_2: hydrogen peroxide; IAA: indole-3-acetic acid. CV: coefficient of variation.

Table S3. Primers sequences and functional annotation of eight candidate genes used for quantitative real–time PCR (qRT–PCR) analysis.

**Gene ID (Encoded protein)**	**Gene position**	**Primer Sequence (5' to 3')**	**Gene functional annotation**
Zm00001d010159 (Actin 1)	Chromosome 8 (102413768–102417536 bp)	F: CGATTGAGCATGGCATTGTCA R: CCCACTAGCGTACAACGAA	ATP binding (GO:0005524); nucleotide binding (GO:0000166); cytoskeleton (GO:0005856)
Zm00001d049601 (Tryptophan decarboxylase 1)	Chromosome 4 (36911317–36913087 bp)	F: CAAGCTCTGGATGGTCATGC R: TGATCCTGAAGCACACGAGA	Aromatic-L-amino-acid decarboxylase activity (GO:0004058); Cellular amino acid metabolic process (GO:0006520); Pyridoxal phosphate binding (GO:0030170)
Zm00001d039346 (Probable indole–3–acetic acid–amido synthetase GH3.12)	Chromosome 3 (2194864–2198030 bp)	F: TCGGTGAGCATTACGAGGTC R: GCTCGCTGTTCTTGTCGATG	Response to light stimulus (GO:0009416); Response to auxin (GO:0009733); Ligase activity (GO:0016874)
Zm00001d026530 (Indole–3–acetic acid–induced protein ARG7)	Chromosome 10 (147742692–147744167 bp)	F: AAGATCCGGTCCATCGTGTG R: GAACCCGTACTCCTCCTCC	Response to auxin (GO:0009733)
Zm00001d049659 (Auxin–induced protein 15A)	Chromosome 4 (38968832–38969823 bp)	F: AGCAGCTGATGACGAGGC R: GAACTCGTCCTCCACCCG	Response to auxin (GO:0009733)
Zm00001d016471 (Trans–cinnamate 4–monooxygenase)	Chromosome 5 (163692318–163694217 bp)	F: CAACTACGGCGACTTCATCC R: CGTTGATGTTCTCGACGATGT	Monooxygenase activity (GO:0004497); oxidoreductase activity, acting on paired donors, with incorporation or reduction of molecular oxygen (GO:0016705)
Zm00001d005998 (Caffeoyl–CoA O–methyltransferase 1)	Chromosome 2 (194954891–194956278 bp)	F: GCAGCTCCTGATCGAGATTC R: GCGACCCTATCTGGTCGTAG	O-methyltransferase activity (GO:0008171); S-adenosylmethionine-dependent methyltransferase activity (GO:0008757); methylation (GO:0032259)
Zm00001d032152 (cinnamoyl CoA reductase1)	Chromosome 1 (214573400–214579274 bp)	F: GCTGCTCGAGAAGGGATACAC R: CGGGTCATCTGCTGCCAT	Circadian rhythm (GO:0007623); response to cold (GO:0009409); lignin biosynthetic process (GO:0009809); cinnamoyl-CoA reductase activity (GO:0016621)
Zm00001d053554 (Peroxidase)	Chromosome 4 (233852931–233854306 bp)	F: GCCCTCTTCGCCTCAGACTT R: TCAGCTGCTGTTGACCACCC	Peroxidase activity (GO:0004601); Response to oxidative stress (GO:0006979); heme binding (GO:0020037)
